# The Cost-Effectiveness of Digitally Supported Mental Well-Being Prevention and Promotion Targeting Nonclinical Adult Populations: Systematic Review

**DOI:** 10.2196/72458

**Published:** 2025-08-11

**Authors:** Sara Claes, Fleur Van De Wielle, Els Clays, Lieven Annemans

**Affiliations:** 1 Department of Public Health and Primary Care, Faculty of Medicine and Health Sciences, Ghent University, C Heymanslaan 10, Ghent, 9000, Belgium, +32 09 332 36 28

**Keywords:** cost-effectiveness, mental health, digital health, health promotion, public health, mobile phone

## Abstract

**Background:**

In recent years, policymakers worldwide have been increasingly concerned with promoting public mental well-being. While digitally supported well-being interventions seem effective in general nonclinical populations, their cost-effectiveness remains unclear.

**Objective:**

This study aims to systematically synthesize evidence on the cost-effectiveness of digitally supported mental well-being interventions targeting the general population or adults with subclinical mental health symptoms.

**Methods:**

PubMed, Embase, Scopus, and Web of Science were systematically searched for health economic or cost-minimization studies. Eligibility criteria included interventions in the general population or adults showing risk factors or subclinical mental health symptoms, with at least 1 digital component. Study quality was comprehensively assessed using the Consensus Health Economic Criteria list.

**Results:**

Of 3455 records identified after duplicate removal, 12 studies were included: 3 studies evaluated universal prevention, 3 investigated selective prevention, and 6 covered indicated prevention. Six studies applied a societal perspective. Incremental cost-utility ratios were reported in 6 of the included studies and varied from dominant to €18,710 (US $ 23,185) per quality-adjusted life year. In general, digitally supported well-being interventions in nonclinical adults, and particularly indicated prevention strategies, seemed to generate improved health outcomes at lower costs from a societal perspective. The quality appraisal highlighted several shortcomings of the available literature.

**Conclusions:**

Overall, the use of digital tools for mental well-being prevention and promotion in nonclinical adult populations has the potential to be cost-effective. Nevertheless, to adequately guide policymaking, more evidence is still needed. Future studies should ensure valid argumentation for the applied time horizon and perspective, alongside rigorous sensitivity analyses in accordance with best practices to improve cost-effectiveness evidence. Furthermore, assessment methods more sensitive to changes in well-being such as the EQ Health and Well-being instrument could be considered.

## Introduction

Over the past decades, policymakers have increasingly recognized that societal welfare goes beyond economic success and includes the mental well-being of citizens as well [1-3]. Mental well-being is commonly conceptualized in terms of three core components: (1) subjective well-being, including life satisfaction, frequent positive emotions, and infrequent negative emotions; (2) positive psychological functioning and self-actualization; and (3) optimal social functioning [4]. The growing interest in public well-being is underpinned by evidence linking mental well-being to a wide range of beneficial outcomes such as greater health and longevity, enhanced productivity, and stronger social relationships [5,6]. Given these far-reaching positive effects, promoting public well-being has become a priority on political agendas worldwide, as reflected in the objectives of the World Health Organization (WHO) and the Council of the European Union (EU Council) [7,8]. The WHO’s Mental Health Action Plan (2013‐2030) prioritizes promotion of mental well-being, prevention of mental ill health, and access to appropriate care, whereas the EU Council advocates for investments in people’s overall well-being as a means to enhance societal resilience and support sustainable economic growth.

The promotion of public well-being could be facilitated with digital tools such as smart devices, mobile apps, and websites, considering their potential for accessibility and scalability. Recently, a meta-analysis indeed found support for the effectiveness of fully automated digital interventions in improving the mental well-being of the general population [9]. Moreover, digital well-being interventions are frequently regarded as a cost-effective strategy [9]. Nevertheless, scientific evidence to support this claim is still limited. A recent systematic review concluded that web-based interventions for mental disorders are likely to be cost-effective compared with care as usual [10]. In addition, evidence suggests that mental health prevention and promotion strategies are cost-effective [11]. However, ambiguity remains about the value for money of digitally supported well-being prevention and promotion for the general public.

**Figure 1.**
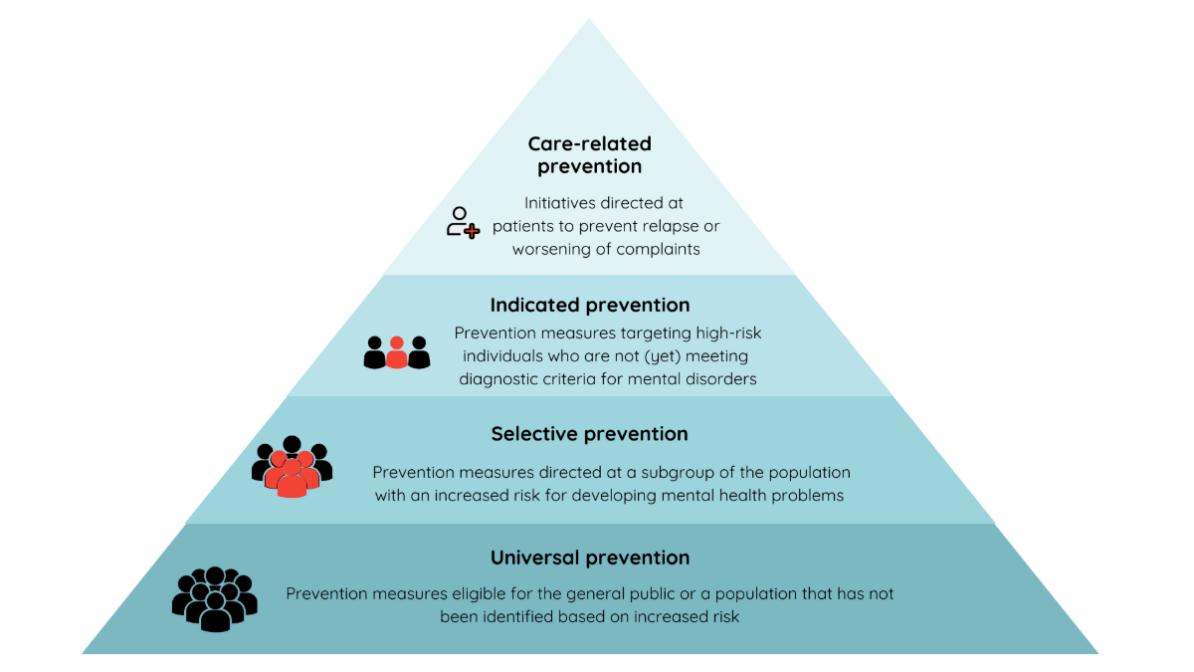
Classification of prevention strategies.

Mental well-being prevention and promotion strategies can be categorized based on their target population. In particular, a distinction can be made between universal, selective, indicated, and care-related prevention (Figure 1) [12,13]. Universal prevention is suitable for the whole population and is directed at the general public, which has not been selected based on specific characteristics or risk factors. In the case of selective prevention, the intervention targets populations with an elevated risk of developing mental health problems. An intervention strategy addressed to individuals identified as high risk, while not meeting diagnostic criteria for mental disorders, is described as indicated prevention. Finally, care-related prevention is directed at patients to reduce the likelihood of relapse and the worsening of complaints and is considered a fundamental part of treatment itself [12,13]. As the focus of this review is on general adult populations, populations at risk, and high-risk adults with up to subclinical symptoms, cost-effectiveness evidence of universal, selective, and indicated prevention strategies with a digital component will be included.

In sum, this study aims to systematically review health economic evaluations and cost-minimization analyses of digitally supported mental well-being interventions intended to prevent mental ill-being and promote mental well-being in the general population, populations with an increased risk, or high-risk adults with up to subclinical symptoms. Since health economic evaluations provide information on both the costs and effects of alternative courses of action, they are of great value to decision makers facing budget constraints. As such, our results will be of interest to policymakers worldwide as they provide information on the value for money of digitally supported well-being prevention strategies. This knowledge is crucial in outlining the optimal strategy for preventing mental health problems and promoting the well-being of citizens.

## Methods

The protocol (PROSPERO: CRD42024516673) and reporting of this systematic review follow the 2020 PRISMA (Preferred Reporting Items for Systematic reviews and Meta-Analyses) guidelines [14] (the PRISMA checklist is provided in Checklist 1).

### Search Strategy

A systematic search was performed in 4 different databases (PubMed, Embase, Web of Science, and Scopus) on February 13, 2024. Email alerts were activated on all databases to receive updates on relevant papers after this date. These email alerts were systematically screened by SC until December 2024. The search strategy included search concepts related to mental well-being, digital interventions, and economic evaluations. The complete search strategy can be consulted in Multimedia Appendix 1.

### Study Selection

The inclusion and exclusion criteria are shown in Textbox 1. The criteria were defined a priori and in line with the population, intervention, comparator, and outcome framework. The aim was to include health economic evaluations or cost-minimization studies on digitally supported mental well-being interventions in nonclinical adult samples, populations at risk, or high-risk adults with subclinical symptoms. Universal, selective, and indicated prevention strategies were thus included. Based on these eligibility criteria, titles and abstracts were screened with Rayyan [15] by 2 independent researchers (SC and FVDW). In a second step, the full texts were screened by 2 reviewers (SC and FVDW) using the same eligibility criteria. Discrepancies were discussed with a third reviewer (LA). To identify potentially missed papers, the reference lists of the included papers were checked.

Textbox 1. **Eligibility criteria for study inclusion.**

**Inclusion criteria:**

- Population: Adults (aged 18 years or older); nonclinical population or at risk for mental issues (up to subclinical symptoms).
- Intervention: Any type of digital well-being intervention including, but not restricted to, digital apps, text messaging, and online website interventions or mixed interventions with both digital and nondigital components.
- Comparator: No intervention, non–digital intervention, same intervention but different frequency.
- Outcomes: Economic outcomes (eg, cost-effectiveness and cost-utility analysis) or cost-minimization outcomes (whereby effects are assumed equivalent across interventions).
- Study design: Model-based or within-trial health economic evaluations.
- Language: English.
- Publication type: Peer-reviewed papers reporting findings of eligible study designs.

**Exclusion criteria:**

- Population: Children, adolescents, animals, and patient populations.
- Intervention: Interventions with no digital component.
- Comparator: Not applicable.
- Outcomes: Outcomes related to effectiveness, intervention costs, or health resource use costs only.
- Study design: Pre/post, reports, systematic reviews, meta-analyses, observational, cross-sectional, case studies, case series, case-control.
- Language: Non-English publications.
- Publication type: Conference abstracts, protocols, commentaries, animal studies, retracted papers, book chapters, and unpublished literature.

### Synthesis of Results

After study selection, the following data from the included studies were extracted: (1) General study characteristics: first author, publication year, country, participant characteristics, type of intervention(s), and comparator. (2) Methods: study perspective, time horizon, type of economic evaluation, and sensitivity analyses. (3) Results: costs, effects, incremental costs, incremental effects, incremental cost-effectiveness ratio (ICER) or incremental cost-utility ratio (ICUR), and author conclusions.

To facilitate comparison across studies, costs and ICERs or ICURs were converted with a web-based calculator to Euro (reference year: 2024 and reference country: Belgium) [16].

### Quality Appraisal

Based on the Consensus Health Economic Criteria list, which is applicable to both trial-based and model-based economic evaluations [17], the quality of the included studies was comprehensively assessed. In line with Werbrouck [18] and Willems et al [19], the Consensus Health Economic Criteria list was slightly adapted. In particular, “not applicable” was considered a valid option when judging discounting (item 14) since discounting applies only to those studies with a time horizon longer than 1 year.

## Results

### Study Selection

**Figure 2.**
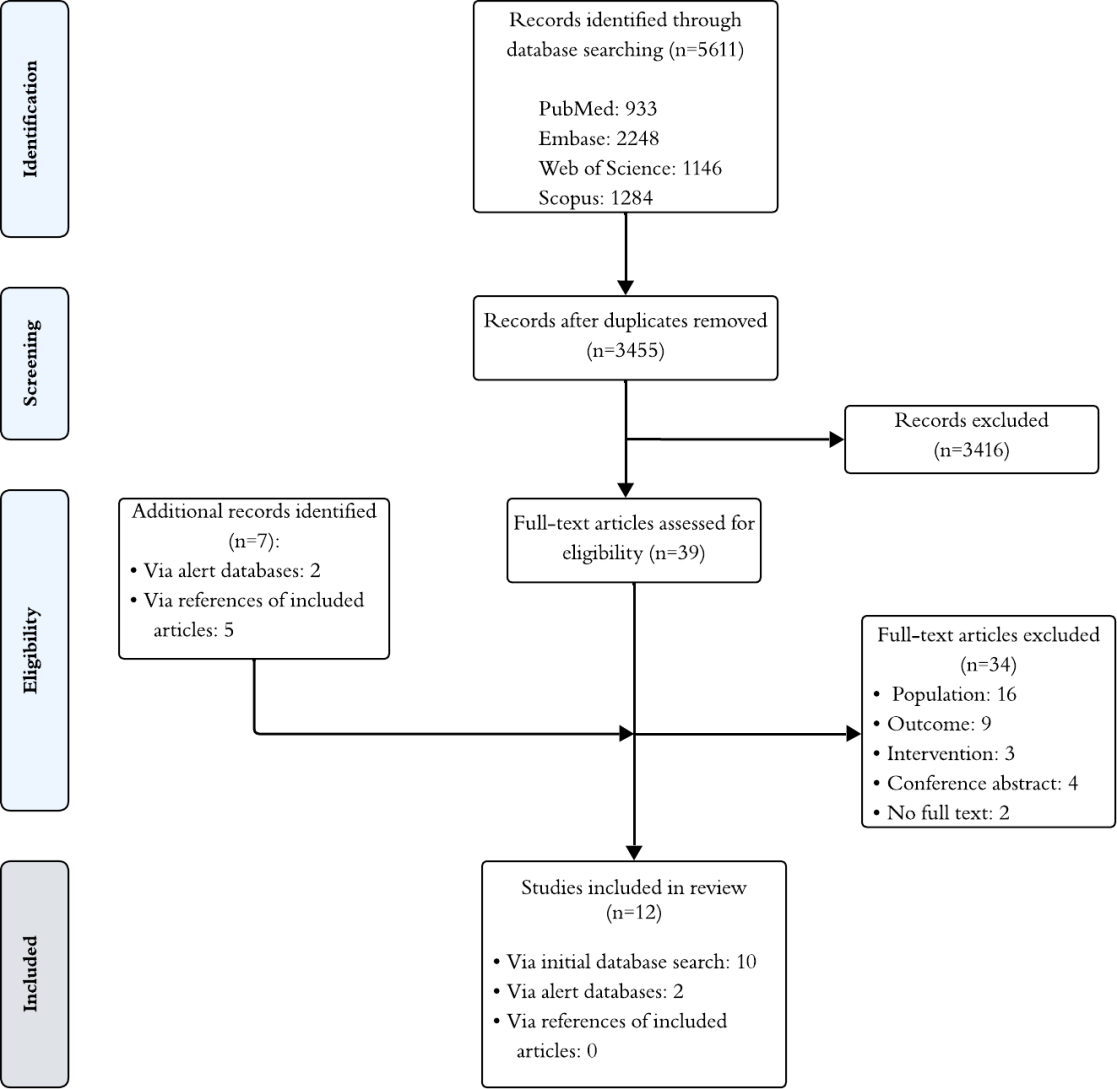
PRISMA (Preferred Reporting Items for Systematic reviews and Meta-Analyses) flowchart for search process and study selection.

An overview of study identification, screening, and inclusion is displayed in the PRISMA flowchart (Figure 2). From 5611 studies identified (3455 after duplicate removal), 12 studies were included in this review after verifying eligibility. Multimedia Appendix 2 [20-53] provides an overview of excluded studies during full-text screening with reasons. Study characteristics and health economic outcomes can be consulted in Table 1. Multimedia Appendix 3 [54-65] provides the full assessment of the studies.

**Table 1.** Evidence table—short.

Study	Country	Prevention type	Intervention	Comparator	Perspective	Time horizon	Results (converted to €, 2024)
Buntrock et al [54]	Germany	Indicated	Web-based	Enhanced CAU^a^	Societal and health care	12 months	ICER^b^ (€ per DFY)^c^ Societal: €1560 (US $1933) Health care: €1571 (US $1947) ICER (€ per QALY^d^) Societal: €18,710 (US $23,185) Health care: €18,851 (US $23,359)
Ebert et al (2018) [55] and Kählke et al [56]	Germany	Indicated	Web-based	WLC^e^	Employer and societal	6 months	Employer: ICER (€ per extra person under symptom threshold) Dominant ROI^f^: 0.61 Societal: ICER (€ per extra person under symptom threshold) Dominant ICER (€ per QALY) Dominant
Fitzhugh et al [57]	United Kingdom	Selective	Application	N/A^g^	Health care^h^	24 weeks	—^i^
Freund et al [58]	Germany	Universal	Web-based	WLC	Societal and employer	6 months	ICER (€ per extra person under symptom threshold) Societal: Dominant Employer: Dominant ICER (€ per QALY) Societal: Dominant Employer: Dominant ROI Employer: 1.77
Le et al [59]	Australia	Indicated	Web-based	No intervention	Partial societal	11 years	ICER (A$/QALY gained) Dominant ROI (A$ saving per A$ invested) 1.05
Lintvedt et al [60]	Norway	Indicated	Web-based	N/A	Health care provider	12 months	aCER^j^ (development costs in € per QALY gained) €4929 (US $6108) ^i^ ROI 8.7
Lokkerbol et al [61]	The Netherlands	Universal	Web-based scenario A: face-to-face preventive interventions + Preventive eHealth interventions Scenario B: A + lower costs	CAU	Health care provider	5 years	ICER A, at 5 years (€ per DALY^k^ averted) €11,361 (US $14,078) ROI A, at 5 years (€ saved by € invested) 1.45 ICER B, at 5 years (€ per DALY averted) €11,279 (US $14,078) ROI B, at 5 years 1.77
Monteiro et al [62] and Monteiro et al [63]	Portugal	Selective and indicated	Web-based	WLC	Societal	14 months	ICER (€ per QALY) Dominant
Noben et al [64]	The Netherlands	Selective	Web-based	No intervention	Societal	6 months	ICER (€ per treatment response) €5400 (US $ 6691)
Schotanus-Dijkstra et al [65]	The Netherlands	Universal	Email-guided	No intervention	Health care	6 months (annualized)	ICER (€ per extra person with flourishing mental health) €1664 (US $2062) ICER (€ per treatment response anxiety) €1458 (US $1807) ICER (€ per treatment response depression) €1584 (US $1963)

a CAU: care as usual.

b ICER: incremental cost-effectiveness ratio.

c DFY: depression-free year.

d QALY: quality-adjusted life year.

e WLC: Wait-list control.

f ROI: return on investment.

g N/A: not applicable.

h Perspective not reported but judged by authors based on available information.

i ICER not reported or calculated correctly.

j aCER: average cost-effectiveness ratio

k DALY disability-adjusted life year.

### Study Characteristics

All but 1 study were conducted in Europe [54-58,60-65]. The remaining study took place in Australia [59]. The studies were published between 2013 and 2024. The majority of studies applied within-trial analyses with a time horizon between 24 weeks and 14 months [54-58,60,62-65]. Six within-trial economic evaluations applied a societal perspective, which includes all health care–related costs regardless of who is paying, as well as costs related to productivity losses [54,56,58,62-64]. Two studies adopted a Markov model to evaluate cost-effectiveness in the long run, with a time horizon of either 5 years [61] or 11 years [59]. One of them applied a health care provider perspective [61], while the other used a partial societal perspective, which did not account for direct nonmedical costs (ie, costs outside the health care sector but directly related to disease) [59]. In both Markov models, all input parameters were literature-driven.

### Interventions

Half of the included studies evaluated an indicated prevention strategy (ie, focus on individuals with an identified elevated risk but not meeting diagnostic criteria) [54-56,59,60,63]. Selective prevention (ie, directed at populations with an increased risk) was assessed in 3 studies [57,62,64], and 3 studies concerned universal prevention strategies (ie, directed at the general public, not selected based on risk factors) [58,61,65].

The value for money of web-based interventions was investigated in 10 studies [54-56,58-63], 1 study evaluated email guidance [65], and the other examined smartphone apps [57]. Most interventions encompassed psychoeducation and exercises [54-56,58-60,62-65]. One study investigated the cost-effectiveness of mindfulness apps [57]. Moreover, 4 studies combined web-based interventions with optional text messages for guidance and support [54-56,58]. Only 1 study examined hybrid prevention (ie, combination of digital and nondigital interventions) [61]. To evaluate value for money, interventions were compared with care as usual [61], enhanced care as usual [54], no intervention [59,64,65], or wait-list control (WLC) [55-58,62,63].

### Well-Being Outcomes

In 7 of the included studies, (incremental) cost per quality-adjusted life year (QALY) was the primary health economic outcome [54,56,58-60,62,63]. Some studies included specific well-being–related outcomes, such as life satisfaction [57], flourishing mental health [65], or treatment response in terms of stress [55,56,58], anxiety [65], or depression [65]. Furthermore, 3 studies documented the return on investment (ROI) [58,59,61].

### Quality Appraisal

The quality appraisal of the included studies is shown in Table 2. Only 2 studies justified the chosen time horizon [59,63]. In the study by Monteiro et al [63], the applied time horizon mirrored the follow-up period of the trial on which health economic evaluation is based, whereas Le et al [59] opted for an 11-year time horizon. These authors argued that a long time horizon is necessary to fully evaluate the impact of preventative strategies. In addition, approximately half of the included studies failed to provide sufficient justification for choosing a perspective other than the societal one [55,57,60,61,65]. Finally, only 6 studies combined probabilistic sensitivity analyses with another type of sensitivity analysis (eg, threshold analysis or one-way sensitivity analysis) on top of the point estimate results and provided adequate argumentation for the applied variable ranges [54,59,62-65].

**Table 2.** Results of quality assessment with the Consensus Health Economic Criteria list.

	Buntrock et al [54]	Ebert et al [55]	Kählke et al [56]	Fitzhugh et al [57]	Freund et al [58]	Le et al [59]	Lintvedt et al [60]	Lokkerbol et al [61]	Monteiro et al [62]	Monteiro et al [63]	Noben et al [64]	Schotanus-Dijkstra et al [65]	Values, %
Study population	1^b^	1	1	0^c^	1	1	0	1	1	1	1	1	83
Competing alternatives	1	1	0	1	0	1	0	1	1	1	1	1	75
Research question	0	1	0	0	1	1	1	1	1	1	1	0	67
Study design	1	1	1	1	1	1	0	1	1	1	1	1	92
Time horizon	0	0	0	0	0	1	0	0	0	1	0	0	17
Perspective	1	0	1	0	1	1	0	0	1	1	1	0	58
Costs: identification	1	1	1	0	1	1	0	1	1	1	1	1	83
Costs: measurement	1	1	1	0	1	1	0	0	1	1	1	0	67
Costs: value	1	1	1	0	1	1	0	1	1	1	1	1	83
Outcomes: identification	1	1	1	1	1	1	0	1	1	1	1	1	92
Outcomes: measurement	1	1	1	1	1	1	1	1	1	1	1	1	100
Outcomes: value	1	1	1	0	1	1	1	1	1	1	1	1	92
Incremental analysis	1	1	1	0	0	1	0	1	1	1	1	1	75
Discounted	N/A^a^	N/A	N/A	N/A	N/A	1	N/A	1	1	0	N/A	N/A	75
Sensitivity analysis	1	0	0	0	0	1	0	0	1	1	0	1	42
Conclusions	1	1	1	0	1	1	0	1	1	1	1	1	83
Generalizability	1	1	1	0	0	1	0	1	1	1	1	0	67
No conflict of interest	1	0	0	1	1	1	1	0	1	1	0	1	67
Ethics	1	1	1	0	1	1	0	1	1	1	0	1	75
Quality score (%)	94	78	73	28	72	100	22	74	95	95	83	72	

a N/A: not applicable.

b Yes.

c No.

### Cost-Effectiveness of Digitally Supported Well-Being Interventions

Overall, the results of the included studies indicate that digitally supported interventions have the potential to cost-effectively promote and support well-being in adults. In particular, for the 6 studies reporting QALYs, all but 1 study found a dominant ICUR, meaning that digital well-being promotion in adults produces better health outcomes while reducing costs [56,58,59,62,63]. In the remaining study, an ICUR of €18,710 (US $23,185) per QALY was found [54].

**Figure 3.**
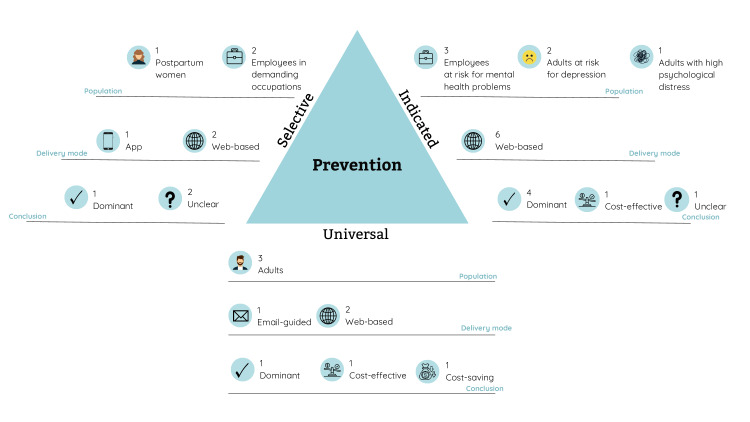
Cost-effectiveness results per prevention type.

Universal prevention strategies with a digital component were found to be cost-effective [65], cost-saving [61], or even dominant (ie, producing better health outcomes at lower costs) [58] (Figure 3). In particular, the ICER in the study by Schotanus-Dijkstra et al [65] equaled €1664 (US $2062) per extra person with flourishing mental health. Lokkerbol et al [61], on the other hand, found an ICUR of €15,764 (US $19,534) per averted disability-adjusted life year or €15,650 (US $19,392) per averted disability-adjusted life year when lowered treatment costs were assumed. The results of these studies appeared to be robust under sensitivity analyses. Nevertheless, only in one of these studies were the evaluations conducted from a full societal perspective instead of a limited perspective [58]. Thus, these cost-effectiveness results should be further confirmed from a societal perspective, providing a broader picture of costs and consequences.

Among the 3 studies evaluating selective prevention strategies, the results were inconclusive (Figure 3). In the study by Monteiro et al [62], web-based cognitive behavioral therapy for women with low risk for postpartum depression appeared to be dominant compared with WLC. Fitzhugh et al [57] also reported favorable results for selective prevention. These authors concluded that mindfulness meditation delivered via smartphone apps is a cost-effective way to promote life satisfaction among police officers. However, the validity of this positive conclusion is limited as it is based on the average cost-effectiveness ratio (ie, the intervention costs divided by the effects of that particular intervention), whereby cost and effects are not compared with an alternative strategy. In a study among nurses, Noben et al [64] evaluated web-based mental health promotion, which was found to be not only less costly but also less effective. Their ICER equaled €5400 (US $6691) per treatment response in terms of improved work functioning. This finding could be related to a lack of integration of the e-mental health tools in the workplace, resulting in reduced effectiveness [64].

Indicated prevention using digitally supported tools appeared to be cost-effective [54], dominant (ie, better health outcomes at lower costs) [55,56,59,63], or cost-saving [60] (Figure 3). However, the cost-saving result in the study by Lintvedt et al [60] should be interpreted with caution since the authors failed to compare the intervention with an alternative, and no sensitivity analyses were performed. Web-based indicated prevention was compared with either WLC [55,56,63] or no intervention [59]. Only Buntrock and colleagues [54] used an active comparator to determine the cost-effectiveness of a guided web-based self-help intervention. This intervention was compared with “enhanced usual care,” which consisted of additional web-based psychoeducation. From a societal perspective, these authors found an ICUR of €18,710 (US $23,185) per QALY.

Despite the lack of comprehensive sensitivity and subgroup analyses in most studies, some cost-effectiveness drivers could be identified. Several studies highlighted costs as key drivers of cost-effectiveness [55,56,61-63,65]. Additionally, 2 studies noted that cost-effectiveness results improved when using more sensitive measures for assessing QALY gains [54,56]. In particular, in both studies, interventions had a higher probability of being dominant when QALYs were measured with the Short Form 6 Dimensions (SF-6D). This measurement tool is more sensitive to changes in mild conditions than the EQ-5D. Furthermore, the modeling assumptions had an impact on the cost-effectiveness findings of Le et al [59]. In particular, the transition probabilities and the assumed duration of intervention effects affected the ROI. However, it is unclear whether these observed changes are statistically significant.

## Discussion

### Principal Findings

Notwithstanding the rapidly growing market for digitally supported mental well-being tools for the general public, their value for money remains unclear [9]. Nevertheless, information on the “best buy” is essential for policymakers as they are faced with the challenge of maximizing citizen well-being while financial resources are scarce [66]. Therefore, this systematic review aimed to summarize the current evidence on the cost-effectiveness of digitally supported interventions to prevent mental ill-being and promote well-being in nonclinical adults. In particular, universal (ie, for the general public), selective (ie, for at-risk populations), and indicated (ie, for individuals with identified vulnerabilities, not meeting diagnostic criteria) prevention strategies with a digital component were included in this review. Alongside offering guidance to policymakers, the results of our review will inform researchers about existing evidence gaps and methodological shortcomings when planning and conducting future health economic evaluations.

Overall, digitally supported interventions appear to cost-effectively promote well-being among nonclinical adult populations. Half of the included studies investigated the cost-effectiveness of digitally supported indicated prevention strategies [54-56,59,60,63], with many reporting dominant results. In particular, these interventions produced better health outcomes at lower costs than WLC [55,56,63] and no intervention [59]. On the other hand, the nondominant, yet cost-effective findings reported by Buntrock et al [54] for web-based indicated prevention could be attributed to their active comparator, which included web-based psychoeducation. Next, digitally supported selective prevention was covered in only 3 studies, and their results were inconclusive. However, due to the limited number of studies, firm conclusions about their cost-effectiveness cannot be drawn. Likewise, evidence on universal prevention strategies with a digital component is sparse. While the available evidence suggests cost-effectiveness, additional research is needed to validate these findings. Finally, whether the mode of delivery (ie, web-based, app, or email) is related to different cost-effectiveness results is hard to evaluate since only 1 study evaluated smartphone apps [57] and 1 study investigated an email-guided intervention [65]. As such, based on the current state of the evidence, identifying a single most cost-effective type of digital prevention strategy for well-being remains challenging, underscoring the need for cautious interpretation and future research.

Despite growing recognition of the importance of public mental well-being, public investments in mental health remain limited, particularly for prevention and promotion. On average, governments across the globe allocate 2.1% of health expenditure to mental health, and in the majority of countries, less than 20% of this share is devoted to the promotion of mental well-being and prevention of mental illness [67]. As such, this review offers timely insights that can inform sustainable public mental health strategies. According to our results, digitally supported tools for well-being promotion in nonclinical adults are generally cost-effective. However, it is important to note that more cost-effectiveness evidence is still needed on digitally supported well-being interventions to adequately guide policymaking. Therefore, we urge policymakers to invest in research on effective and cost-effective well-being promotion and to make decisions informed by such research.

Based on the findings of this review, several recommendations for future research can be formulated as well. First, as mentioned before, cost-effectiveness evidence on digitally supported well-being promotion and prevention remains limited. Health economic evaluations should become standard practice when conducting interventional research. Additionally, to improve assumptions made and reduce uncertainty regarding long-term intervention effects, trials should encompass longer follow-up periods. Second, when planning future economic evaluations, researchers are advised to carefully consider outcome measurement. According to 2 studies, cost-effectiveness results improved when using more sensitive measures for assessing QALYs [54,56]. In the general population or people with subclinical symptoms, the EQ-5D might suffer from ceiling effects, potentially leading to an underestimation of effectiveness and cost-effectiveness results [68]. Additionally, only 1 of 5 EQ-5D items measures mental health. To address these limitations of the EQ-5D, future research could consider assessment methods more sensitive to changes in well-being, such as the EQ Health and Well-being (EQ-HWB) instrument, which was specifically designed for use in health economic evaluations and captures both health and well-being [69]. Likewise, the WELLBY (well-being year) approach could offer a more comprehensive assessment of consequences in economic evaluations for well-being promotion initiatives [66]. Compared with the QALY, the WELLBY has a broader outlook and defines quality of life in terms of subjective well-being or how people feel about their lives. As a single-item measure, the WELLBY can easily complement existing preference-based measures of health, such as the EQ-5D, in health economic evaluations. Third, the ICUR or ICER should be interpreted in light of a willingness-to-pay (WTP) threshold. Two of the included studies [57,60] did not report a WTP threshold, while a range of different thresholds was applied in the remaining studies. For prevention, WTP thresholds are indeed less well-established, and there are valid arguments for both higher and lower thresholds compared with those for treatment [70]. Additionally, future research should investigate the WTP threshold for well-being outcomes (eg, EQ-HWB, WELLBY, depression-free years, etc). Given the limited consensus on WTP thresholds for well-being and preventive measures, researchers are encouraged to report their results in light of a WTP threshold accompanied by valid argumentation for the applied threshold. The WHO recommends a threshold of 1-3 times the gross domestic product per capita [71]. However, in light of opportunity costs, a more conservative threshold of 50% of the gross domestic product per capita has been proposed as well [72,73]. Fourth, there is abundant room for further progress in determining cost-effectiveness drivers. For instance, it is unfortunate that none of the studies explored the impact of dropout and adherence on cost-effectiveness results. This should be addressed in future economic evaluations since digital interventions are likely to suffer from reduced adherence [74].

Furthermore, our quality appraisal highlighted several shortcomings of the available literature, which should be addressed in future research. First, in most studies, justification for the applied time horizon was lacking. Future studies are thus advised to choose a time horizon that captures relevant long-term costs and effects and to articulate the rationale behind their choice clearly. Second, the study’s perspective determines which costs are considered in the economic evaluation and thus affects cost-effectiveness results. Since different stakeholders are involved in and affected by health promotion and prevention, it is recommended to apply a full societal perspective or even multiple perspectives [75]. In any case, transparency about the perspective used and the underlying rationale is key. Third, sensitivity and subgroup analyses were insufficiently conducted in several studies, limiting the robustness of the results and our understanding of cost-effectiveness drivers. To deal with data uncertainty and underlying assumptions in health economic evaluations, future studies should adopt best methodological practices, such as conducting sensitivity analyses and subgroup analyses according to current standards [17]. For instance, when conducting sensitivity analyses, clear justification for the applied variable ranges should be provided.

### Strengths and Limitations

A major strength is that this review provided a comprehensive overview of the value for money of digitally supported well-being promotion for adults, with a particular focus on universal, selective, and indicated prevention, to conclude with clear recommendations for both policymakers and researchers. Nevertheless, some limitations should be addressed. First, although evidence on the effectiveness of digital tools for well-being promotion and prevention is growing, the number of cost-effectiveness studies remains low, particularly for universal and selective prevention. As such, it was impossible to thoroughly compare different prevention strategies (ie, universal, selective, and indicated) in terms of cost-effectiveness to properly guide policymaking. Second, because of methodological variation, comparison across the different studies should be done with caution. Third, due to the limited number of health economic evaluations and cost-minimization studies, publication bias could not be comprehensively assessed. It is indeed possible that cost-effective results are favored. Therefore, as health economic evidence accumulates, this should be further explored.

### Conclusions

This systematic review summarizes the current cost-effectiveness evidence for digitally supported well-being promotion for adults. In particular, universal, selective, and indicated prevention strategies are included. Overall, such interventions appear to be cost-effective, with several studies showing dominance. As such, digital tools could offer a financially sustainable approach to promoting public mental well-being. Nevertheless, the evidence available remains low. Future studies are needed to address evidence gaps as well as existing methodological shortcomings by choosing an appropriate time horizon that encompasses all relevant costs and consequences, improving transparency, and conducting sufficient sensitivity analyses to account for data uncertainty. Furthermore, assessment methods more sensitive to changes in well-being, such as the EQ-HWB or WELLBY approach, could be considered.

## Supplementary material

10.2196/72458 Multimedia Appendix 1 Search terms.

10.2196/72458 Multimedia Appendix 2 Reasons for exclusion at the full-text stage.

10.2196/72458 Multimedia Appendix 3 Evidence table full.

10.2196/72458 Checklist 1 PRISMA (Preferred Reporting Items for Systematic reviews and Meta-Analyses) checklist.

## Abbreviations

EQ-HWB: EQ Health and Well-being
EU Council: Council of the European Union
ICER: incremental cost-effectiveness ratio
ICUR: incremental cost-utility ratio
PRISMA: Preferred Reporting Items for Systematic reviews and Meta-Analyses
QALY: quality-adjusted life year.
ROI: return on investment
SF-6D: Short Form 6 Dimensions
WELLBY: well-being year
WHO: World Health Organization
WLC: wait-list control
WTP: willingness-to-pay
