# Supplementary material for: The Cost-Effectiveness of Digitally Supported Mental Well-Being Prevention and Promotion Targeting Nonclinical Adult Populations: Systematic Review
Source: JMIR Ment Health. 2025 Aug 11;12:e72458. doi: 10.2196/72458 (PMC12369993; doi:10.2196/72458)
Supplement: Multimedia Appendix 1 [file mental-v12-e72458-s001.docx]

A1: Search terms

Pubmed

(“happiness”[tiab] OR “happy”[tiab] OR “wellbeing”[tiab] OR “well-being”[tiab] OR “flourish*”[tiab] OR “life satisfaction”[tiab] OR “satisfaction with life”[tiab] OR "positive affect"[tiab] OR "negative affect" [tiab] OR "positive emotion*" [tiab] OR "negative emotion*"[tiab] OR "mental health"[MeSH] OR "mental health"[tiab] OR "psychological well-being"[MeSH])

AND

("telemedicine"[MeSH] OR "telemedicine"[tiab] OR "mobile health"[tiab] OR “mhealth”[tiab] OR "m-health"[tiab] OR "internet" [tiab] OR "online" [tiab] OR "internet-based" [tiab] OR "web-based" [tiab] OR "webbased" [tiab] OR "e-health" [tiab] OR "ehealth" [tiab] OR "telehealth" [tiab] OR "tele-health" [tiab] OR "digital" [tiab] OR “smartphone”[MeSH] OR “smartphone*”[tiab] OR “mobile phone*”[tiab] OR “mobile applications”[MeSH] OR "mobile application*"[tiab] OR "app"[tiab] OR "apps" [tiab])

AND

(“Cost effectiveness analysis” [MeSH] OR “Cost-Effectiveness analysis” [MeSH] OR “Cost-Benefit Analysis” [MeSH] OR “Cost effectiveness analy*” [Title/Abstract] OR “Cost-effectiveness analys*” [tiab] OR “Costeffective*” [tiab] OR “Costefficien*” [tiab] OR “Cost effective*” [tiab] OR “Cost-effective*” [tiab] OR “Cost Effectiveness Ratio” [tiab] OR “Incremental cost-effectiveness ratio” [tiab] OR “Incremental cost effectiveness ratio” [tiab] OR “Cost-Effectiveness Ratio” [tiab] OR “Cost-Benefit Analy*” [tiab] OR “Cost Benefit Analy*” [tiab] OR “Costutility Analy*” [tiab] OR “Cost utility Analy*” [tiab] OR “Cost-Utility Analy*” [tiab] OR “Cost utility” [tiab] OR “Cost-utility” [Title/Abstract] OR “Costutility” [tiab] OR “Cost Benefit” [tiab] OR “Costbenefit” [tiab] OR “Costs and Benefits” [tiab] OR “Benefits and Costs” [tiab] OR “Cost-Benefit Data” [tiab] OR “Cost Benefit Data” [tiab]OR “cost analy*” [Title/Abstract] OR "costanaly*" [tiab] OR “Economic Evaluation*” [tiab] OR "health economic*"[tiab] OR "healtheconomic*"[tiab] OR “Value for money” [tiab] OR “Cost efficiency analy*” [tiab] OR “Cost benefit assessment*” [tiab] OR "cost-benefit assessment*"[tiab] OR “Cost benefit ratio” [tiab] OR "cost-benefit ratio"[tiab] OR “Cost benefit evaluation*” [tiab] OR "cost-benefit evaluation" [tiab] OR "cost minimization" [tiab] OR "costminimization" [tiab] OR "quality adjusted life year*" [tiab] OR "qaly*"[tiab] OR "quality adjusted*" [Title/Abstract] OR "qualityadjusted*" [tiab])

NOT

((("Adolescent"[MeSH Terms] OR "Child"[MeSH Terms] OR "Infant"[MeSH Terms] OR "adolescen*"[Title/Abstract] OR "child*"[Title/Abstract] OR "schoolchild*"[Title/Abstract] OR "infant*"[Title/Abstract] OR "girl*"[Title/Abstract] OR "boy"[Title/Abstract] OR "teen"[Title/Abstract] OR "teens"[Title/Abstract] OR "teenager*"[Title/Abstract] OR "youth*"[Title/Abstract] OR "pediatr*"[Title/Abstract] OR "paediatr*"[Title/Abstract] OR "puber*"[Title/Abstract]) NOT ("Adult"[MeSH Terms] OR "adult*"[Title/Abstract] OR "man"[Title/Abstract] OR "men"[Title/Abstract] OR "woman"[Title/Abstract] OR "women"[Title/Abstract])) OR "review"[PT] OR "systematic review"[PT] OR "meta-analysis"[PT] OR "editorial"[PT] OR "practice guideline"[PT] OR "case reports"[PT] OR "clinical conference"[PT] OR "congress"[PT] OR "validation study"[PT])

= 933 (13/02/2024)

Embase

('happiness'/exp OR 'happiness':ti,ab,kw OR 'happiness':ti,ab,kw OR 'happy':ti,ab,kw OR 'wellbeing':ti,ab,kw OR 'well-being':ti,ab,kw OR 'flourish*':ti,ab,kw OR 'life satisfaction':ti,ab,kw OR 'satisfaction with life':ti,ab,kw OR 'positive affect':ti,ab,kw OR 'negative affect':ti,ab,kw OR 'positive emotion*':ti,ab,kw OR 'negative emotion*':ti,ab,kw OR 'psychological well-being'/exp OR 'mental health'/exp OR 'mental health':ti,ab,kw)

AND

('telemedecine'/exp OR 'telemedicine':ti,ab,kw OR 'telehealth'/exp OR 'telehealth':ti,ab,kw OR 'tele-health':ti,ab,kw OR 'mobile health':ti,ab,kw OR 'mhealth':ti,ab,kw OR 'm-health':ti,ab,kw OR 'internet':ti,ab,kw OR 'online':ti,ab,kw OR 'internet-based':ti,ab,kw OR 'web-based':ti,ab,kw OR 'webbased':ti,ab,kw OR 'e-health':ti,ab,kw OR 'ehealth':ti,ab,kw OR 'digital':ti,ab,kw OR 'smartphone'/exp OR 'mobile phone'/exp OR 'mobile app*':ti,ab,kw OR 'app':ti,ab,kw OR 'apps':ti,ab,kw)

AND ('health economics'/exp OR 'economic evaluation'/exp OR 'cost benefit analysis'/exp OR 'cost effectiveness analysis'/exp OR 'cost utility analysis'/exp OR 'cost minimization analysis'/exp OR 'cost effectiveness analy*':ti,ab,kw OR 'cost-effectiveness analys*':ti,ab,kw OR 'costeffective*':ti,ab,kw OR 'costefficien*':ti,ab,kw OR 'cost effective*':ti,ab,kw OR 'cost-effective*':ti,ab,kw OR 'cost effectiveness ratio':ti,ab,kw OR 'incremental cost-effectiveness ratio':ti,ab,kw OR 'incremental cost effectiveness ratio':ti,ab,kw OR 'cost-effectiveness ratio':ti,ab,kw OR 'cost-benefit analy*':ti,ab,kw OR 'cost benefit analy*':ti,ab,kw OR 'costutility analy*':ti,ab,kw OR 'cost utility analy*':ti,ab,kw OR 'cost-utility analy*':ti,ab,kw OR 'cost utility':ti,ab,kw OR 'cost-utility':ti,ab,kw OR 'costutility':ti,ab,kw OR 'cost benefit':ti,ab,kw OR 'costbenefit':ti,ab,kw OR 'costs and benefits':ti,ab,kw OR 'benefits and costs':ti,ab,kw OR 'cost-benefit data':ti,ab,kw OR 'cost benefit data':ti,ab,kw OR 'cost analy*':ti,ab,kw OR 'costanaly*':ti,ab,kw OR 'economic evaluation*':ti,ab,kw OR 'health economic*':ti,ab,kw OR 'healtheconomic*':ti,ab,kw OR 'value for money':ti,ab,kw OR 'cost efficiency analy*':ti,ab,kw OR 'cost benefit assessment*':ti,ab,kw OR 'cost-benefit assessment*':ti,ab,kw OR 'cost benefit ratio':ti,ab,kw OR 'cost-benefit ratio':ti,ab,kw OR 'cost benefit evaluation*':ti,ab,kw OR 'cost-benefit evaluation*':ti,ab,kw OR 'cost minimization':ti,ab,kw OR 'costminimization':ti,ab,kw OR 'quality adjustd life year*':ti,ab,kw OR 'qaly*':ti,ab,kw OR 'quality adjusted*':ti,ab,kw OR 'quality adjusted':ti,ab,kw))

NOT

((('adolescent'/exp OR 'child'/exp OR adolescent*:ti,ab,kw,kw OR child*:ti,ab,kw OR schoolchild*:ti,ab,kw OR infant*:ti,ab,kw OR girl*:ti,ab,kw OR boy*:ti,ab,kw OR teen:ti,ab,kw OR teens:ti,ab,kw OR teenager*:ti,ab,kw OR youth*:ti,ab,kw OR pediatr*:ti,ab,kw OR paediatr*:ti,ab,kw OR puber*:ti,ab,kw ) NOT ('adult'/exp OR 'aged'/exp OR 'middle aged'/exp OR adult*:ti,ab,kw OR man:ti,ab,kw OR men:ti,ab,kw OR woman:ti,ab,kw OR women:ti,ab,kw)) OR ('review'/exp OR 'systematic review'/exp OR 'meta-analysis'/exp OR 'editorial'/exp OR 'practice guideline'/exp OR 'case report'/exp OR 'conference paper'/exp OR 'validation study'/exp))

= 2248 (13/02/2024)

Web of Science

(TS=("happiness" OR "happy" OR "wellbeing" OR "well-being" OR "flourish*" OR "life satisfaction" OR "satisfaction with life" OR "positive affect" OR "negative affect" OR "positive emotion*" OR "negative emotion*" OR "mental health")

AND

TS=("telemedicine" OR "mobile health" OR "mhealth" OR "m-health" OR "internet" OR "online" OR "internet-based" OR "web-based" OR "webbased" OR "e-health" OR "ehealth" OR "telehealth" OR "tele-health" OR "digital" OR "smartphone*" OR "mobile phone*" OR "mobile application*" OR "app" OR "apps")

AND TS=("cost effectiveness analy*" OR "cost-effectiveness analy*" OR "costeffective*" OR "costefficien*" OR "cost effective*" OR "cost-effective*" OR "cost effectiveness ratio" OR "incremental cost-effectiveness ratio" OR "incremental cost effectiveness ratio" OR "cost-effectiveness ratio" OR "cost-benefit analy*" OR "cost benefit analy*" OR "costutility analy*" OR "cost utility analy*" OR "cost-utility analy*" OR "cost utility" OR "cost-utility" OR "costutility" OR "cost benefit" OR "costbenefit" OR "costs and benefits" OR "benefits and costs" OR "cost-benefit data" OR "cost benefit data" OR "cost analy*" OR "costanaly*" OR "economic evaluation*" OR "health economic*" OR "healtheconomic*" OR "value for money" OR "cost efficiency analy*" OR "cost benefit assessment*" OR "cost-benefit assessment*" OR "cost benefit ratio" OR "cost-benefit ratio" OR "cost benefit evaluation*" OR "cost-benefit evalation*" OR "cost minimization" OR "costminimization" OR "quality adjusted life year*" OR "qaly*" OR "quality adjusted*" OR "qualityadjusted*"))

NOT

(TS=("adolescen*" OR "child*" OR "schoolchild*" OR "infant*" OR "girl*"OR "boy"OR "teen" OR "teens" OR "teenager*" OR "youth*" OR "pediatr*" OR "paediatr*" OR "puber*") OR AK=("review" OR "systematic review" OR "meta analysis" OR "meta-analysis" OR "trial protocol" OR "study design" OR "study protocol" OR "editorial" OR "practice guideline" OR "clinical guideline" OR "case report" OR "case-report" OR "conference paper" OR "validation study"))

= 1146 (13/02/2024)

Scopus

TITLE-ABS-KEY({happiness} OR {happy} OR {wellbeing} OR {well-being} OR "flourish*" OR {life satisfaction} OR {satisfaction with life} OR {positive affect} OR {negative affect} OR "positive emotion*" OR "negative emotion*" OR {mental health})

AND

TITLE-ABS-KEY({telemedicine} OR {mobile health} OR {mhealth} OR {m-health} OR {internet} OR {online} OR {internet-based} OR {web-based} OR {webbased} OR {e-health} OR {ehealth} OR {telehealth} OR {tele-health} OR {digital} OR {smartphone} OR "mobile phone*" OR "mobile application*" OR {app} OR {apps})

AND

TITLE-ABS-KEY("cost effectiveness analy*" OR "cost-effectiveness analy*" OR "costeffective*" OR "costeffecien*" OR "cost effective*" OR "cost-effective*" OR {cost effectiveness ratio} OR {incremental cost-effectiveness ratio} OR {incremental cost effectiveness ratio} OR {cost-effectiveness ratio} OR "cost-benefit analy*" OR "cost benefit analy*" OR "costutility analy*" OR "cost utility analy*" OR "cost-utility analy*" OR {cost utility} OR {cost-utility} OR {costutility} OR {cost benefit} OR {costbenefit} OR {costs and benefits} OR {benefits and costs} OR {cost-benefit data} OR {cost benefit data} OR "cost analy*" OR "costanaly*" OR "economic evaluation*" OR "health economic*" OR "healtheconomic*" OR {value for money} OR "cost efficiency analy*" OR "cost benefit assessment" OR "cost-benefit assessment*" OR {cost benefit ratio} OR {cost-benefit ratio} OR "cost benefit evaluation*" OR "cost-benefit evaluation*" OR {cost minimization} OR {costminimization} OR "quality adjusted life year*" OR "qaly*" OR "quality adjusted*" OR "qualityadjusted*")

AND NOT

TITLE-ABS-KEY ( "adolescen*" OR "child*" OR "schoolchild*" OR "infant*" OR "girl*" OR "boy" OR "teen" OR "teens" OR "teenager*" OR "youth*" OR "pediatr*" OR "paediatr*" OR "puber*" AND NOT ( "adult*" OR "man" OR "men" OR "woman" OR "women" ) )

AND NOT

KEY ( {review} OR {systematic review} OR {meta analysis} OR {meta-analysis} OR {trial protocol} OR {study protocol} OR {study design} OR {editorial} OR {practice guideline} OR {clinical guideline} OR {case report} OR {case-report} OR {conference paper} OR {validation study} )
